# *C. elegans srf-6* and *nsy-1* mutations result in a similar 2AWC^ON^ phenotype and do not complement (*srf-6* is *nsy-1* II)

**DOI:** 10.17912/micropub.biology.000128

**Published:** 2019-07-04

**Authors:** Brooke E. Honzel, Stephen J. Foley, Samuel M. Politz

**Affiliations:** 1 Department of Biology and Biotechnology, Worcester Polytechnic Institute, Worcester, MA; 2 Department of Chemistry and Biochemistry, Worcester Polytechnic Institute, Worcester, MA

**Figure 1.**
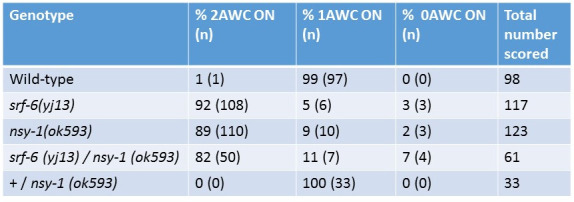
**Figure 1**. Comparison of AWC phenotypes of *srf-6(yj13)* and *nsy-1(ok593)* mutants, and complementation testing of *srf-6(yj13)* and *nsy-1(ok593)* mutants using the 2AWC^ON^phenotype. The *nsy-1(ok593)* mutation is a large complex rearrangement that completely deletes the catalytic domain of *nsy-1* (*C. elegans* Deletion Mutant Consortium 2012). Homozygous *srf-6* and *nsy-1* mutants also contained homozygous *unc-4 (e120) II* and *kyls140 I.* Table entries represent percentages of worms with each phenotype, followed by the actual number of worms scored in parentheses. The right-hand column indicates the total number of worms scored per genotype.

## Description

*C. elegans srf-6* mutants were isolated using altered surface immunofluorescence as phenotype (Hemmer et al., 1991; Grenache et al., 1996). In Van Sciver et al., 2019, we showed by whole genome sequencing that three different *srf-6* mutants carry mutations in gene *nsy-1.* Well-characterized mutant alleles of *nsy-1* result in hermaphrodites that express the olfactory receptor gene *str-2* in both AWC neurons (2AWC^ON^, Troemel et al., 1999), unlike wild type hermaphrodites, which express *str-2* asymmetrically in only one of the two AWC neurons (1AWC^ON^, Troemel et al., 1999). Our sequencing results suggested that *srf-6* mutants might have a similar 2AWC^ON^phenotype. We therefore examined *srf-6(yj13)* for its AWC phenotype.

In order to introduce an *str-2::GFP* marker into the *srf-6* genotype, first *srf-6(yj13) unc-4(e120) II and nsy-1(ok593) unc-4(e120)* II double mutants were constructed as described (Hemmer et al., 1991). Homozygous males carrying the construct *kyIs140*, which contains an *str-2::GFP* fusion integrated on chromosome I (Troemel et al., 1999), were mated with *srf-6(yj13) unc-4(e120) II* hermaphrodites, and an Unc F2 hermaphrodite expressing GFP in chemosensory neuron AWC was cloned. An individual hermaphrodite descendant, all of whose offspring expressed GFP, was isolated to establish a strain of genotype *kyls140 I; srf-6(yj13) unc-4(e120) II.* A strain of genotype*kyls140 I; nsy-1(ok593) unc-4(e120)* was constructed similarly. Adult hermaphrodites from these strains were examined in a fluorescent microscope for their AWC phenotype. Figure 1 shows that *srf-6(yj13)* adults exhibited a 2AWC^ON ^phenotype similar to that of *nsy-1(ok593).*

To test whether *srf-6(yj13)* and *nsy-1(ok593)* affect the same or different genetic functions, a complementation test was performed (Figure 1, last two lines). Males of genotype *srf-6(yj13)* were mated with *kyls140 I; nsy-1(ok593) unc-4(e120) II* hermaphrodites, and non-Unc offspring were scored in a fluorescent microscope for the AWC phenotype. The complementation heterozygotes showed a 2AWC^ON ^phenotype similar to that of *nsy-1(ok593). * This result included data from two separate crosses. In contrast, when wild type males were mated with *kyls140 I; nsy-1(ok593) unc-4(e120) II* hermaphrodites, the 1AWC^ON^phenotype was observed. These results included worms from one cross.

These results indicate that s*rf-6(yj13)* and *nsy-1(ok593)* mutations do not complement each other for the 2AWC^ON ^phenotype, and together with the *srf-6* mutant sequencing results (Van Sciver et al., 2019), we conclude that *srf-6* and *nsy-1* are the same gene.

## Reagents

*C. elegans* Strains

N2 *C. elegans* wild type **

CX3695 *kyIs140* [*str-2*::GFP + *lin-15*(+)] I

AT18 *srf-6(yj13)* II

AT19 *srf-6(yj13) unc-4(e120)* II

VC390 *nsy-1(ok593)* II

AT28: *srf-6(yj13) unc-4(e120)* II; *kyIs140* [*str-2*::GFP + *lin-15*(+)] I

AT29: *nsy-1(ok593) unc-4(e120)* II

AT30: *nsy-1(ok593) unc-4(e120)* II; *kyIs140*[*str-2*::GFP + *lin-15*(+)] I

Strains N2, CX3695, and VC390 are available from the CGC. Strains AT28 and AT30 will be submitted to the CGC.
